# Clinical Informatics Team Members’ Perspectives on Health Information Technology Safety After Experiential Learning and Safety Process Development: Qualitative Descriptive Study

**DOI:** 10.2196/53302

**Published:** 2024-02-05

**Authors:** Chantelle Recsky, Kathy L Rush, Maura MacPhee, Megan Stowe, Lorraine Blackburn, Allison Muniak, Leanne M Currie

**Affiliations:** 1 School of Nursing University of British Columbia Vancouver, BC Canada; 2 School of Nursing University of British Columbia Okanagan Kelowna, BC Canada; 3 Digital Health Provincial Health Services Authority Vancouver, BC Canada; 4 Vancouver Coastal Health Vancouver, BC Canada; 5 Health Quality BC Vancouver, BC Canada

**Keywords:** informatics, community health services, knowledge translation, qualitative research, patient safety

## Abstract

**Background:**

Although intended to support improvement, the rapid adoption and evolution of technologies in health care can also bring about unintended consequences related to safety. In this project, an embedded researcher with expertise in patient safety and clinical education worked with a clinical informatics team to examine safety and harm related to health information technologies (HITs) in primary and community care settings. The clinical informatics team participated in learning activities around relevant topics (eg, human factors, high reliability organizations, and sociotechnical systems) and cocreated a process to address safety events related to technology (ie, safety huddles and sociotechnical analysis of safety events).

**Objective:**

This study aimed to explore clinical informaticians’ experiences of incorporating safety practices into their work.

**Methods:**

We used a qualitative descriptive design and conducted web-based focus groups with clinical informaticians. Thematic analysis was used to analyze the data.

**Results:**

A total of 10 informants participated. Barriers to addressing safety and harm in their context included limited prior knowledge of HIT safety, previous assumptions and perspectives, competing priorities and organizational barriers, difficulty with the reporting system and processes, and a limited number of reports for learning. Enablers to promoting safety and mitigating harm included participating in learning sessions, gaining experience analyzing reported events, participating in safety huddles, and role modeling and leadership from the embedded researcher. Individual outcomes included increased ownership and interest in HIT safety, the development of a sociotechnical systems perspective, thinking differently about safety, and increased consideration for user perspectives. Team outcomes included enhanced communication within the team, using safety events to inform future work and strategic planning, and an overall promotion of a culture of safety.

**Conclusions:**

As HITs are integrated into care delivery, it is important for clinical informaticians to recognize the risks related to safety. Experiential learning activities, including reviewing safety event reports and participating in safety huddles, were identified as particularly impactful. An HIT safety learning initiative is a feasible approach for clinical informaticians to become more knowledgeable and engaged in HIT safety issues in their work.

## Introduction

### Background

Health care delivery is increasingly dependent on technology, and health care organizations are heavily investing in technological infrastructure [1]. Health information technologies (HITs), such as electronic health records (EHRs), computerized provider order entry, and mobile devices, play an ever-increasing role in clinical practice, and it is largely thought that these technologies have the potential to promote safe care and contribute to better patient outcomes [2,3]. However, a growing body of research [4-7] has also identified the potential for HITs to contribute to harm, where harm is defined as something “that should not have happened and that you don’t want to happen again” [8]. In this study, the acronym HIT refers to technologies used in the health care system for health information management. This study focused on HIT safety concerns, more specifically, unintended harm or potential harm that involved an HIT-based system.

An example of harm related to HIT is an overdose event that occurred when a patient aged 16 years received 39 antibiotic pills when they should have received only 1 pill [9]. The overdose event occurred after a series of computer and human failures, including a confusing computer interface design that forced weight-based dosing for pediatrics, an automated “robot” system in the pharmacy that performed a “double check” rather than human verification, a hidden curriculum for prescribing doctors to “ignore all computer alerts,” and a novice nurse who trusted the computer recommendations because “the computer had been right” in the past [9]. The design of technology and overreliance on the accuracy of the information presented can lead to unintended consequences, and there is increasing recognition that events such as this are occurring alongside the increasing uptake of technologies in health care [4-6,10-13]. There is also a growing identification that well-designed and well-deployed systems may help to mitigate some of these issues [14,15]. Clinical informatics teams, with expertise in the effective use of HITs, are ideally positioned to recognize and respond to HIT-related harms and contribute toward enhanced quality and safety in health care delivery. In this study, we explored clinical informaticians’ experiences in incorporating safety practices into their work.

### Defining HIT Safety and Harm

The field of patient safety has conventionally focused on hospital and inpatient settings, with the measurement and monitoring of adverse events (including near misses) well established as a widespread practice designed to reduce harm from activities in the acute care setting (eg, medications, surgical procedures, falls, and diagnostics) [16-19]. For this study, focused on primary and community care settings, a broader view of safety was taken up, with consideration for all 6 interconnected dimensions of quality health care (safety, effectiveness, patient centeredness, timeliness, efficiency, and equity) [20]. Using the broad definition of harm as “something happened that you did not want to happen,” harm is not simply the opposite of safety; instead, the definition makes space for recognizing the potential for harm related to other circumstances, such as inequity, inaccessibility, and poor patient experiences. Recognizing the interconnectedness of the 6 dimensions of quality, an expansive definition of harm is useful because it places a greater emphasis on the complex nature of the health care system and can thus help identify latent problems. A focus on latent problems that contribute to harm (as opposed to a focus on human error) has the potential to yield more systems-focused solutions to mitigate against recurrence and thereby improve the quality of care. This is particularly relevant with HIT, where latent errors may impact people who are several degrees away from an HIT origin of error, such as the numerous clinicians involved in the overdose example described in the preceding section.

### Embedded Research Context

This study was conducted as part of an embedded researcher project supported by an innovative program and funding model designed to maximize the impact of research by supporting formal partnerships between emerging academics and health system leaders to address pressing challenges in health service delivery [21]. The funding program provided support for a doctoral student researcher to conduct their dissertation research while holding a position within the health service organization. The program is designed to help researchers develop professional skills to support evidence-informed improvement within the health system alongside conventional research outputs [22]. The embedded researcher, the researcher’s supporting academic committee, and leaders from the organization collaborated to design an applied research project that met usual academic requirements and simultaneously was relevant and useful to the organization. In project conceptualization, the health care organization prioritized patient safety and a focus on HIT in primary and community care settings. Upon funding, the researcher was embedded within the organization’s community clinical informatics team for a 2-year period (2019 to 2021). The embedded researcher was experienced in clinical education and had experience in the areas of clinical nursing, clinical education, informatics, patient safety, and quality improvement. Having a role within the organization allowed the researcher extraordinary insights into the role of the team within the organization, the team’s learning needs related to HIT safety, and opportunities to address learning needs. Fostering daily working relationships was an intentional aspect of the project to support engagement and build capacity in the team, apply learning to existing processes, and promote sustainable practices [23]. The organization’s quality and safety leaders were also key contributors to the project, collaborating closely with the embedded researcher to guide the course of the project.

### Codevelopment of the HIT Safety Process

In the early stages of the project, the embedded researcher worked with a clinical informatics leader to conduct a retrospective sociotechnical analysis of reported HIT safety concerns [24]. This part of the project was possible because the organization had added a question (“Was a computer involved in the incident?”) to their web-based voluntary incident reporting system in 2016. The findings from the incident analysis study [25] provided the foundation for the clinical informatics team and the embedded researcher to cocreate a new process that uses sociotechnical systems analysis for identifying, analyzing, and responding to HIT safety events. The new process used safety huddles [26,27] and was aligned with the concept of a learning health system in which theoretical frameworks and scientific evidence are integrated with internal data to inform continuous improvements [28,29]. Throughout the project, the researcher was positioned within the clinical informatics team with a constant focus on facilitating learning using adult learning principles [30-32], drawing on a variety of fields such as patient safety [17,18,20,33,34], quality improvement [35-37], and learning health systems [38-40] as well as HIT safety and harm specifically [5,41-45]. The aim of this study was to examine the clinical informaticians’ experiences in learning about HIT safety and to understand their experiences in codeveloping the new process to address HIT safety concerns in their work.

## Methods

### Setting

**Figure 1:**
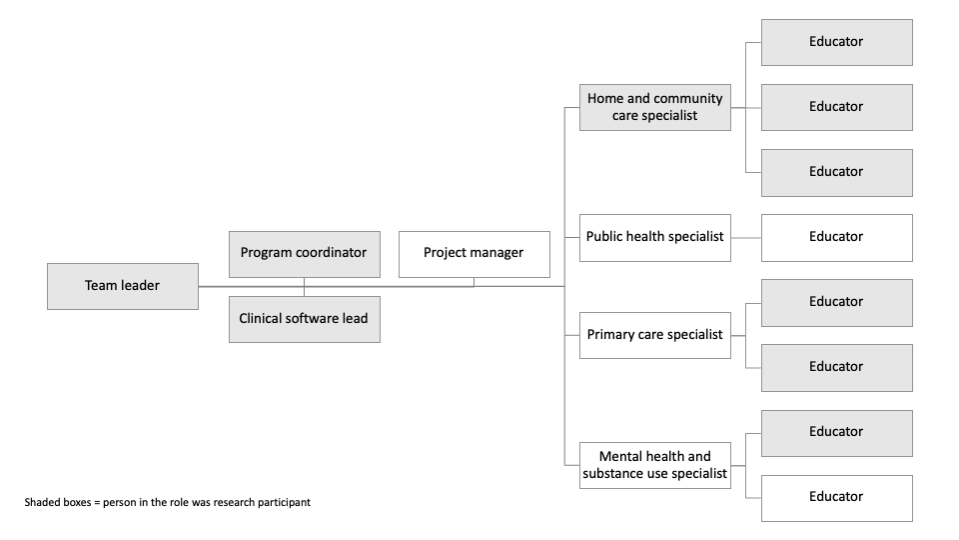
Team chart and research participation.

The study was carried out in a large health care organization in western Canada that provides acute, primary, and community care for >1.25 million people and includes both densely populated urban areas as well as rural and remote communities. The study focused on members of a clinical informatics team assigned to support services delivered in non–acute care settings (ie, primary care, home care, population and public health, long-term care, mental health, and substance use services). The clinical informatics team comprised 15 to 20 multidisciplinary staff members as part of the organization’s efforts to strengthen primary and community care delivery. The team was established at the beginning of the research project, which proved to be serendipitous for the research project because the embedded researcher was able to enter a newly formed group. The team was responsible for the clinical integration and operation of all HIT systems in primary and community care settings. There were different roles and responsibilities among the members of the team. For example, the educators were responsible for supporting clinical staff in using the clinical software systems. The specialists were responsible for working with the HIT software development team to communicate the changes that might be required for the HIT system (eg, practice policy changes). In addition, team members were dedicated to specific clinical service areas, such as mental health or home care. The clinical informatics team did not include any prescribing clinicians, pharmacists, or medical office assistants; however, the embedded researcher did consult and obtain input from members of these groups to inform them of the learning experiences and codevelopment of the HIT safety process. An organizational chart of the team is available in Figure 1.

### Study Design

This study used qualitative description methods to explore the clinical informaticians’ perspectives and reflections on their experiences of learning about HIT safety and the codevelopment of the process to manage HIT safety events. Qualitative description is useful for capturing the meanings and interpretations that informants ascribe to their experiences [46,47]. Aligned with a constructivist paradigm, which posits that human knowledge is subjective and socially constructed, the study aimed to capture both the participants’ perspectives and acknowledge the subjectivity and involvement of the embedded researcher in constructing interpretations of the data [48]. In this study, having been embedded within the team for an extended period, the researcher was able to glean an in-depth understanding of the context surrounding the participants’ accounts.

During data collection and analysis, the embedded researcher engaged in ongoing reflexivity by attending to their unique positioning, the circumstances surrounding the study, and the potential influences on knowledge construction [48]. From a postpositivist perspective, the close relationship between the researcher and participant is conventionally thought to perpetuate bias and prevent the attainment of rigor in research [49,50]. However, we contend that it was the strength of the relationship between the embedded researcher and the clinical informatics team that allowed the researcher to fully explore how HIT safety was in alignment (or not) with the work of the team. The intention of situating an embedded researcher with the team was to encourage relationships between the researcher and the clinical informatics team members to support the meaningful, effective, and sustainable integration of knowledge into practice [51-54]. Indeed, as team members began to apply their learning in their work (ie, using the sociotechnical framework to analyze a problem), the researcher was available for guidance and consultation as needed, and as the team’s capabilities developed, less support from the researcher was required.

### Ethical Considerations

Before conducting this study, ethics approval was obtained from the University of British Columbia Research Ethics Board (H18-02677), and all participants signed a consent form. The conduct and reporting of the study followed the Consolidated Criteria for Reporting Qualitative Studies guidelines for qualitative research reporting.

### Data Collection

We used purposive sampling, targeting clinical informatics team members who supported primary and community care and who had participated in the HIT safety initiatives over the previous 12 to 24 months. The embedded researcher emailed all team members (N=16), inviting them to participate in 1 of 3 web-based focus groups, with a clear statement that participation was optional and in no way related to their job. The study was carried out during the COVID-19 pandemic when face-to-face meetings were discouraged; thus, the embedded researcher facilitated focus groups over Zoom (Zoom Video Communications, Inc) [55]. A semistructured interview guide was used (Multimedia Appendix 1), and the focus group sessions were recorded using Zoom video capture, downloaded onto the private secure computer of the researcher, and manually transcribed verbatim by the researcher. The informants reviewed the transcript of their comments, and all participants approved the transcripts with no revisions.

### Data Analysis

Thematic analysis was conducted [46,56,57] using NVivo (version 12; Lumivero). Thematic analysis is well suited to address broad research questions and provides a flexible approach to remain “data-near” [47], searching across the data for patterns and allowing for both inductive and deductive approaches to the analysis. An overarching framework of 3 categories—barriers, enablers, and outcomes (Textbox 1)—was used to provide an initial structure for the analysis.

After completing the deductive coding to classify the data as barriers, enablers, or outcomes, the data were re-examined to inductively generate subcategories. Subcategories were developed and refined over several iterations (between CR and LMC), and a member-checking session was conducted with the clinical informatics team to support the descriptive and interpretive validity of the findings [46,48].

**Operational definitions of high-level analytic categories.**

- Barriers to understanding and applying methods to address health information technology (HIT) safety: Activities or conditions that may have impeded learning
- Enablers to learning about safety and mobilizing their knowledge: Activities or experiences that facilitated or promoted learning
- Outcomes of the HIT safety project: A product or result from engaging in learning activities

## Results

### Overview

Three 1-hour web-based focus groups were held with 10 informants. Of the 10 informants, half were in clinical informatics educator roles, and the other half held roles such as clinical informatics team leader or clinical informatics project manager. A total of 50% (5/10) of the informants had been in their current role for <2 years, 40% (4/10) for 2 to 5 years, and 10% (1/10) for >10 years. In total, 90% (9/10) of the informants had a clinical background, including 6 nurses, 1 physiotherapist, 1 occupational therapist, and 1 social worker. The team member, who did not have a clinical background, had been working in the health care sector for >10 years. The informants’ previous work experience in their respective clinical roles before taking on an informatics-focused role ranged from 0 to 24 years, with an average of 9.7 years.

**Figure 2:**
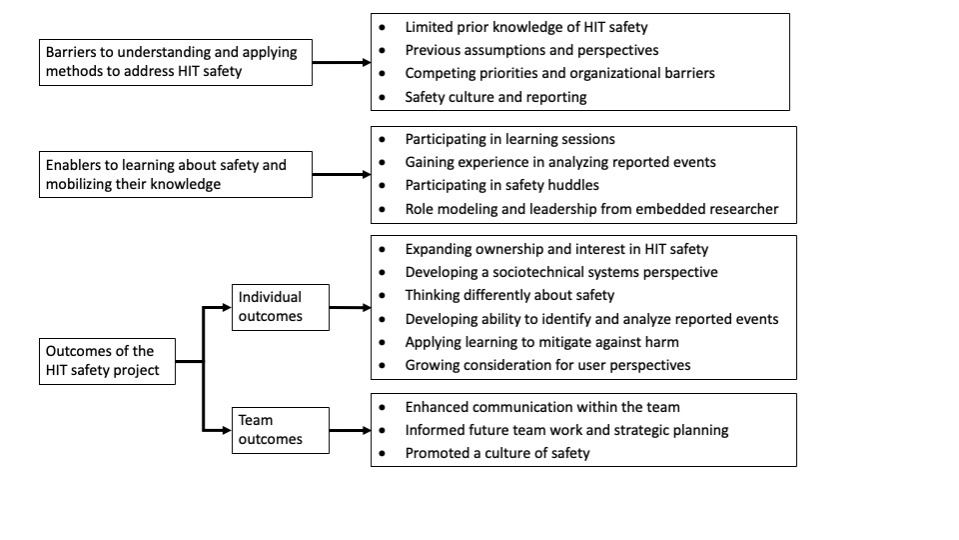
Overview of the results. HIT: health information technology.

The informants shared several barriers and enablers related to their experiences of learning about and developing strategies to address safety. They also described outcomes such as new learning and capabilities. Figure 2 displays the categories and subcategories. A description of each item follows, including excerpts from the data. Characteristics of the individual informants are limited to preserve their anonymity.

### Barriers to Understanding and Applying Methods to Address HIT Safety

The informants identified several barriers to understanding and applying methods to address HIT safety, including a lack of knowledge, previously held perspectives, organizational pressures, and challenges related to event reporting before the project began.

### Limited Prior Knowledge of HIT Safety

A common barrier described by the informants was initially having little or no foundational knowledge related to safety principles and their application to HIT. As an informant reported, “My experience with technology safety was pretty limited prior to all of this.... I didn’t really know much at all.” More specifically, informants shared that they had a limited understanding of any negative impact HIT systems could have on patient care. For example, one of the most experienced informants stated, “I had no idea what was going on for people at the front-line level with whatever they consider a computer incident.” This limited initial understanding exemplifies the learning needs within the team that needed to be addressed to begin to incorporate HIT safety practices into their work.

### Previous Assumptions and Perspectives

The informants also shared different assumptions and perspectives about safety and harm in health care that they had previously held. Some were unaware that HIT could introduce risks to safety, were under the impression that safety concerns were beyond the scope of their role, or attributed safety concerns to user mistakes. An informant stated:

Technology was always supposed to be something that would make things safer, right? You streamline some processes, you make some things maybe, more automatic, take out some of the human element—that should be safer.

Others expressed that they were initially uncertain whether this topic was relevant. For example, an informant who had been with the team for 2 years stated, “I felt like it wasn’t really my role or responsibility to be identifying them [safety concerns].” Another shared that they had previously assumed another department of “internal auditors” was responsible for addressing problems related to HIT and now they saw the value in having a clinical perspective, “focusing on solutions, rather than, it’s just a number.”

Some informants described their initial attitudes toward reported HIT safety events as “narrow,” “judgey,” or “blaming,” articulating that they previously dismissed these events as user error. An educator described this shift away from a perspective of blame as, “not just dismissing it as ok, this one person screwed up this one thing, but more than likely, that person isn’t the only one who’s maybe made that same mistake.” Another educator explained how their perspectives moved from blaming toward curiosity:

I came from a narrow point of view, you [the end user] did something wrong, the [HIT] system didn’t work for you, what was that about? And now it’s more about, more curious—what else happened? How could we support you? What would’ve filled the gaps to make this less of an incident?

### Competing Priorities and Organizational Barriers

The informants indicated that their capacity and capabilities to address HIT-related safety concerns were challenged by competing priorities and organizational barriers. An informant who had worked within the organization for 4 years perceived this as a systems issue: “The whole organization gets caught up in all these new initiatives, all these new projects, all these things you know to make things better, but learning from the past and all these safety incidents, I feel like they get brushed aside.” Another team member described recognizing that there was a need for a focus on HIT safety but struggled with knowing how to proceed: “It just seemed too big to wrap our hands around without some support.”

### Safety Culture and Reporting

A further challenge was related to the internal patient safety reporting system and processes, including concerns that it is onerous to use and, therefore, underutilized. A person who had extensive clinical experience questioned the usefulness of the reporting system, “Coming from the mindset of a clinician, you’re busy you know, am I going to take the time to do a [report], what’s the value there?”

On a broader scale, there were also knowledge gaps related to using the existing reporting system. As 1 person commented, “There’s still a lot to do in developing the reporting culture around technology needs in community*.*” More specifically, an informant noted the point-of-care staff’s lack of understanding of what constituted a computer-related safety event, “People don’t really understand what [an HIT] system-related error really is, and so a lot gets put into that [reporting] system that may not be appropriate for our eyes.”

The informants were also concerned that underuse of the patient safety reporting system meant that issues reported in the event reports were just the tip of the iceberg: “There’s so many safety issues that we don’t know about, things that are actually happening that aren’t being reported.” The challenges with the reporting system impeded their ability to address HIT issues because fewer reports meant that they had fewer opportunities for learning by analyzing events. Another informant made an analogy to the concurrent COVID-19 pandemic:

It’s just like the COVID out there right now, there’s probably more cases than there actually are, we just don’t know about them...what we don’t know, is how to actually properly capture that all, and encourage people to come forward when they have an issue.

### Enablers to Learning About HIT Safety and Mobilizing Their Knowledge

The informants reported some key enablers to learning about HIT safety and mobilizing their knowledge, including making space to participate in learning sessions and safety huddles, the hands-on experience of analyzing reports, and their observations of the embedded researcher as a role model.

### Participating in Learning Sessions

Team members identified short education sessions led by the embedded researcher as supportive of their learning, having “collapsed all the salient points into a quick, easy-to-understand, salient presentation.” The evidence and resources referenced in the learning sessions were also identified as helpful: “I don’t know that there’s often opportunity to bring in scientific literature into our day-to-day jobs so I think that was a great opportunity, to hear and to see what’s happening in the academic realm and consider its application to practice.”

The informants specifically highlighted the case study about the antibiotic overdose (described earlier in this paper) [9] from the learning sessions as an effective tool to understand complexity and sociotechnical systems. A clinical informatics educator who had been with the organization for 3.5 years stated: “That [case study] was very, very engaging and very interesting, and you could see how like, just to see the breakdown like that...and it again, made me aware of all these little things that can go wrong or have to go wrong to lead to something like this, and how the [HIT] system played into it at each step.”

### Gaining Experience Analyzing Reported Events

The informants also shared that the experience of analyzing safety events was valuable in learning how to apply what was gleaned from the analyses to make improvements. An informant who was newer to the team explained, “Actually receiving the [reports] and actually doing the investigative work teaches you a lot about the [HIT] systems.... I enjoyed doing that and then thinking about how it could be better in the long run.” They went on to explain how this experience provided an opportunity to consider how different sociotechnical dimensions may be related to safety concerns: “You’re not just thinking about the actual documentation system, but you’re thinking about all the systems around that, like whether it be a workflow, or chaos, or whatever it is that contributed to that scenario.”

### Participating in Safety Huddles

The informants also explained that the experience of sharing the event analyses with other members of the team and participating in the safety huddles was supportive of their learning around HIT safety in that this activity provided a peer learning experience. One of the educators stated, “I found [safety huddles] really informative, especially having other people there that use other [HIT] systems, and again, it’s someone else’s perspective and how they’re reading the situation and what I can learn from that other person that I’m working with.”

Another team member expressed an appreciation for safety huddles as a venue for communication among the team members and found them worth the time and effort for the team:

I found that making space for us, like dedicated time and focus, to talk about these concerns that we have, or the patient safety events that have occurred.... I’d never had that experience before, and I found it was so helpful talking as a group about what we found or what those problems that were being reported were about.... I found that it was easy to actually make the time and spend the effort to do that. You know we are all busy, but I think in the long run it’s all going to do us well as an organization and as a team to continue that

the safety huddles

### Role Modeling and Leadership From the Embedded Researcher

The informants also indicated that the role of the embedded researcher supported their learning and facilitated changes within the team’s practices. An informant who had been with the team for 2 years described the key function of the embedded researcher as initiating a focus on HIT safety: “I think we needed someone to come in and really help to set the tone and set that framework...and so it’s been learning. I think the last year has been learning across the board.” Building on this, the informants also recognized the embedded researcher as an expert and champion for HIT safety, as a different informant explained: “It’s helped really mobilize the team in that direction and create more of a team sort of focus on working with these teams committed to safety-related issues*.*” An educator from the team described the embedded researcher as a role model for how to approach analyzing safety events, having “instilled curiosity” in the team*.* Finally, there was also a recognition of how to integrate HIT safety into the work of the informatics team; a long-standing member of the team noted, “what I didn’t realize before is really how well this conversation fits within an organizational structure and within a team structure.”

### Outcomes

A variety of outcomes from the HIT safety project surfaced in the focus group discussions, some of which were individual outcomes and some of which were team outcomes. Individual outcomes included increased ownership and interest in HIT safety, the development of a sociotechnical systems perspective, and increased consideration of end-user perspectives. Team outcomes included increased team communication and the ability to use the processes to guide strategic planning.

### Individual Outcomes

#### Expanding Ownership and Interest in HIT Safety

Several informants shared an increased sense of personal interest in the topic of HIT safety. A team member explained, “It’s given me an appreciation and actual interest in safety and how that pertains to design of [HIT] systems and how we interact with the [HIT] systems.” Furthermore, an informant with 8 years at the organization described having an increased sense of both personal and team ownership in relation to HIT safety: “Not only is it my role and responsibility, but we’re really well positioned to identify and sort of bridge between practice and workflows.” The informants recognized the role their team plays within the organization in supporting the delivery of care, as one person with extensive clinical and informatics experience noted, “Having a good understanding of why we exist as informatics...it’s not just for the users, although that’s important, it’s also for the patients and reducing risk...so it all ties together...that kind of holistic view.”

#### Developing a Sociotechnical Systems Perspective

The informants shared new insights into their work based on learning about sociotechnical systems theory as it applies to informatics. They expressed an increased appreciation for the relationships among the technology, the users, and the context in which these are situated. An educator with 24 years of experience explained:

I sort of think that technology doesn’t take into account the human being. It’s just, technology is a set of algorithms, it’s a set of stuff that’s written by a developer who tries to take in all the considerations possible. But you can’t take in all the considerations of a human being, and how a human being will respond to certain situations, or certain pieces of technology.

This was echoed by another educator with 3.5 years of experience, who stated, “Our technology is only as good as how people understand it, and so the education piece around it and you know, understanding the workflow and how to actually apply it and use our [HIT] systems is so important...because the [HIT] system could be working as designed, but if people don’t know how to use it properly, then it just leads to a lot of problems.”

Related to this, the notion of human factors as it applies to safe HIT use was also a new concept for many team members. An informant explained, “I never knew this existed, human factors—and now I am seeing that there is a whole theory behind it, there’s a lot to learn about, there’s best practices in design, there’s all these things that I had no idea even existed.”

#### Thinking Differently About Safety

Building on their knowledge of the sociotechnical perspective, the informants demonstrated an increased awareness of the factors and circumstances that may increase the risks of harm. As one educator highlighted, they previously “had just kind of considered the obvious errors, like with a malfunction or with a bug or something like that.” However, this educator went on to explain how their awareness of safety risks had grown beyond the technical aspects of HIT, noting, “Technology’s not infallible. There’s so many factors, and it’s quite complex, and it’s given me kind of an appreciation for...the whole topic, and it makes me think about problems in a different way.” Similarly, another educator described how their view of safety had expanded beyond just focusing on the HIT end user:

It’s not simply, one person did something wrong. There [are] so many different things—there’s the workflow, the human factor, was it the actual user interface—all those different subcategories.

#### Developing the Ability to Identify and Analyze Reported Events

The informants shared how they applied what they learned to their work and developed the ability to identify and analyze HIT-related safety events. First, among the informants, there was a greater awareness of “just what is a safety event,” as one of the more experienced members of the team put it. Several people described adopting the practice of systematically analyzing reported events. For example, how they learned to “think of [HIT safety events] in those different dimensions that we learnt about with the sociotechnical model, just being able to, like, think of it in a framework like that, in sort of a structured way, to help break it down.” Another informant shared how taking a systematic approach had changed their thinking:

It does help us see sort of where perhaps a gap was with a reported [event]. So, before we just knew there was an issue, but...I wasn’t looking at it as all these different sort of levels.

Similarly, another informant explained:

What I’ve learned is how to break it up. Was it the [HIT] system?... Was it the workflow? The process?

#### Applying Learning to Mitigate Against Harm

Going a step further, informants also articulated how they learned to be more proactive and tried to prevent harm from occurring in the future. An educator explained that they have observed patterns over time from the analyses of events, and this has increased their awareness of the potential for future risks:

I’m actually already starting to see some patterns and starting to think about the complexities of some of these [HIT] systems. Just this one tiny little move can make a big change, can put someone at risk, and it shouldn’t be that easy to put a client [patient] at risk.

Another educator described having an increased awareness of patterns as well, and how this informed their thinking around mitigating future issues: “I think being aware of the patterns and being aware of the common sort of issues is really helpful in terms of thinking about future solutions and making sure that those problems can’t be easily replicated.” Furthermore, the informants described how they had been able to communicate concerns about HIT safety in the context of their work. One of the more experienced team members explained, “I didn’t always have the language to describe why something in the [HIT] system was a problem, but that information with systems thinking really helped me frame those conversations.” Another informant described how the clinical informatics team has begun to take a more proactive approach to safety:

I think it’s also changed the conversation around; just when we’re discussing [HIT] system changes or potential projects that we may undertake, is just the safety risks factors. Having more general dialogue around that...just being more proactive.

#### Growing Consideration for User Perspectives

Consideration for clinicians and HIT users was a commonly expressed sentiment. Team members reflected on their previous experiences as clinical care providers and reflected on this when considering the functions and dysfunctions of HIT in a clinical context. An educator commented:

When we get these reports now, I’m trying to think like the clinician. I’m not working as a clinician anymore, but I am trying to put myself in their shoes—what are all these other surrounding factors, what led them to report about this?

Another informant in the role of educator elaborated on this idea:

But it’s probably easier if you have the open mind to actually really understand, again the empathy factor of it, understanding what had happened in terms of if it’s a system error or whatever, and yeah, it’s just having that understanding that it’s not always the person at fault, it’s not always the system at fault, it could be a combination of everything. And again, what do we do next? It’s how do we learn from this.

Pairing this consideration with their knowledge of HIT safety, another informant demonstrated new insight into the users’ experiences with HIT:

And so they think it’s one way, and then an error happens because they misread something or they didn’t know to check somewhere, and...I just felt like I never clued into how much the design can really impact that front end user experience.

Furthermore, another informant contextualized the challenges clinicians and HIT users may face in using HIT:

I’m just thinking about, really, the environment that people are working in and the complexity of that...the environment might be...very chaotic, and then we’re asking them to do something very complex in the [HIT] system. I think that is a safety concern.... I think that’s where it would be very easy to have errors, obviously.

### Team-Level Outcomes

#### Enhanced Communication Within the Team

The informants described how sharing their experiences about HIT safety worked to enhance communication within the team and further expand their awareness of potential HIT safety concerns. As described earlier, there were separate groups within the team that focused on different HIT systems and clinical areas in primary and community care, which could sometimes create siloed communication. Team members expressed that participating in the HIT safety activities opened up new communication channels and supported an exchange of learning across the different teams. An educator, whose work was focused on a particular clinical area, shared the following:

It was great awareness to hear what was happening in other places...kind of raising that awareness so that if we see something similar in our clinical area, it just kind of alerts you to look out for things that you maybe would’ve never considered...and all of a sudden, your level of awareness is there.

Another informant highlighted how increasing communication across the smaller teams within the larger clinical informatics team helps provide better support to the clinicians or HIT users: “Clinicians are interacting with many systems, and many applications, and many types of technologies, that in fact, we need those opportunities to speak with our colleagues who lead or support other [HIT] programs so that we really get a sense of what those safety events meant.”

#### Informed Future Teamwork and Strategic Planning

On a broader scale, the informants also gleaned new insights into the role of their team within the organization. One of the more senior team members asserted:

I think that our team is perfectly positioned to handle, be handlers of technology related [safety] reports, and make sure we close the loop. I think it needs to be part of our work and just have it as a regular ongoing piece of work that we do and...service that we provide to the organization.

In addition, the informants shared insights into how their knowledge of HIT safety relates to organizational decision-making and strategic planning. An informant in an educator role considered how their team can contribute to future decisions about HIT: “Whether it be just the organization, or operationally within a clinic, and they make a request for a change in a [HIT] system, or it’s a bigger change, like made at a higher level, even at the [executive] level, maybe they’re not making the best decision because we’re not providing them with the best information about our clinical [HIT] systems.”

#### Promoted a Culture of Safety

From a patient safety perspective, the informants shared an increased emphasis on a culture of safety and leveraged learning from their analyses to mitigate future concerns and make improvements. An informant explained:

I think as a team member in clinical informatics, instead of focusing on the mistakes...thinking of the next time. What can be done better? How can it be resolved much better? It’s always the next time, it’s always learning, and it’s always a different situation. But it’s probably easier if you have the open mind to actually really understand...it’s just having that understanding that it’s not always the person at fault, it’s not always the system at fault, it could be a combination of everything. And again, what do we do next? How do we learn from this?

## Discussion

### Principal Findings

#### Overview

This study examined clinical informaticians’ perspectives on learning about HIT safety and the cocreation of a process to manage HIT-related safety reports. To our knowledge, this is a novel examination of the topic. The findings from this study provide valuable new insights into the barriers and facilitators to developing HIT safety within clinical informaticians’ practices, as well as the potential outcomes that a robust, evidence-based approach to knowledge mobilization can have.

#### Barriers to the Uptake of HIT Safety

From the clinical informaticians’ perspectives, some of the barriers that were initially challenging included the lack of knowledge about HIT safety and previous assumptions they carried at the outset of the project. For patient safety in general, a lack of knowledge about incident reporting and assumptions about the value or repercussions of event reporting are known barriers to initiating reports [58]. The embedded researcher was able to spend time at the beginning of the project to build relationships and assess the team’s gaps in knowledge. Recognizing the team’s initial limited understanding of HIT safety, learning activities were focused on fundamental topics in HIT safety and contextualizing learning within their existing work. Another identified barrier was competing priorities and organizational barriers, a challenge echoed in the literature about evidence-based practice [51,59]. While competing priorities may be a perennial challenge in health care, strategically aligning and incorporating learning activities into the current priorities of the clinical informatics team was crucial because mutual learning and appreciation of others’ perspectives and contributions may lead to better processes and outcomes by generating more relevant and applicable knowledge [60].

The findings surfaced challenges with the internal safety reporting systems, the processes, and the underuse of the safety system. The informants expressed concern over the value of reporting if there is not a proper follow-up, stressing the value of “closing the loop” to ensure that the person who reported the event is aware of the implications of their report from an informatics perspective. The costs and benefits of safety reporting are debated in the literature. Insufficient action following a safety report is thought to negatively affect clinicians’ commitment to the reporting process [61]. Macrae [62] argues that “we collect too much and do too little” [63], explaining that although the technical infrastructure for safety reporting has been established in many health care organizations, the requisite processes of investigation and improvement have been underemphasized. Other research suggests that low rates of safety reporting derive from clinicians being prone to applying quick fixes or workarounds to system failures rather than reporting issues to trigger more in-depth analysis and sustainable solutions [64,65]. In any case, it seems that safety reporting is yet to achieve its full potential [61], and the latency of safety issues related to HIT may pose further challenges, with near misses and errors being dismissed or going undetected [66]. However, the findings of this study suggest a way to establish practices to identify and mitigate latent errors.

#### Supportive Learning Environment and Openness to New Ideas

The enabling elements identified by the clinical informaticians focused primarily on participatory and experiential activities. Facilitating informal, locally owned processes for clinical informaticians’ learning around safety has been shown to enable the staff to raise concerns and actively contribute to improvement [58]. Although there is a growing collection of research studies that have applied the Sittig and Singh [24] sociotechnical model in analyzing safety concerns [27,67-69], no literature was identified where this framework was incorporated into experiential learning activities or embedded into clinical informatics work processes in real time. Safety huddles are thought to be transformational in shifting attitudes and practices related to safety, providing a “reliable framework for interdisciplinary communication and action” [26]; however, the evidence to support this is largely anecdotal [70]. Menon et al [27] used safety huddles to address EHR safety concerns in a hospital setting, and although the format differed from this project, the huddles promoted a culture of safety for clinical informaticians, providing a venue for open communication about safety concerns and facilitating learning and improvement, which was also found in this study.

The informants also noted the role the embedded researcher played on the team to “set the tone,” “mobilize the team,” and “instill curiosity,” which supported their learning in the project. A concerted effort was made in the initial stages of the project to develop strong, collaborative partnerships at all levels of the organization [52,60]. At the end of the project, efforts were made to promote the sustainability of the cocreated sociotechnical analysis of HIT safety events by integrating new practices into existing team workflows, and the researcher intentionally stepped back to allow the clinical informatics team to carry out new practices with minimal support [71].

The findings indicated that the clinical informatics team exhibited the knowledge, skills, and attitudes of an effective team [72], which in turn support the notion of a high reliability team [73]. For example, the participants found the safety huddles a “good use of their time.” Furthermore, formal financial and organizational support for the embedded researcher throughout the duration of the project created a fertile environment for learning, with dedicated resources, endorsement and collaboration from leaders, and multiple opportunities for interactions between researchers and knowledge users [51,60].

#### Effective Knowledge Mobilization

The findings of this study include positive outcomes in terms of moving knowledge into action. The focused approach to supporting HIT safety seemed to support a group-level identity transformation, incorporating different professional perspectives, adding value, and acting as a lever for system-wide, evidence-informed sustainable change [74]. The informants described being more knowledgeable and engaged in HIT safety issues in their work. They developed their knowledge base in clinical informatics, with an increased recognition of some perennial problems related to HIT [60,75], and the contextual issues that surround the use of HIT in health care settings [76,77]. The informants also expressed greater ownership regarding safety. This is echoed in the literature in which EHR safety is ascribed as being a shared responsibility among key stakeholders including EHR developers, health care organizations and users, and government regulators [42]. Clinical informatics, with its emphasis on bridging technical and clinical perspectives, can play a central role in facilitating efforts to improve safety [78,79].

The cocreated sociotechnical analysis process for addressing HIT safety events produced immediate and ongoing insights to inform operational decision-making within the organization. The current findings provide additional evidence that a clinically focused informatics team is well positioned to take on this work of “closing the loop” with the end user using the system to report an event. Similarly, by leveraging voluntary safety reporting for quality assurance, Williams et al [54] identified 242 EHR-related safety events analyzed by nurse informaticians, 30 of which led to specific system changes to improve usability.

In this study, informants expressed an appreciation for the structure that the Sittig and Singh [24] analytic framework brought to conducting an analysis and that they learned to look for patterns in reviewing reported events. The process developed by the team was an adaptive approach based on experiential cycles of learning, from which they gradually developed new insights and expanded their collective expertise on HIT safety [80]. Demonstrating a thoughtful approach to safety, the findings indicate that informants’ perspectives moved beyond a reactive “find and fix” approach and instead they were embracing complexity to “enable things to go right more often” [81].

The findings of our study also indicate that enhanced communication helped team members develop a more empathetic approach to supporting clinicians using HIT. Specifically, safety huddles were thought to have improved communication within the team as well as informed their perspectives on all aspects of their work, including planning for future HIT-related needs of the organization [27,82]. Although safety event reporting is not without its limitations [83], reporting can effectively contribute to participatory learning, improve practice, and promote safer care [84].

### Limitations

A possible limitation of this study is that the embedded researcher functioned as both the lead of the initiative and the interviewer for this project. It is possible that the informants’ responses were influenced by social desirability [48]. However, the participants’ responses also showed vulnerability. For example, several respondents indicated that they had been judgmental about end users’ errors in the past. This level of candor suggests that social response bias may have been minimal and possibly was overcome by prolonged engagement, given the long duration that the embedded researcher participated with the team. This study also assessed only the experiences of the clinical informaticians in retrospect. It did not account for the impact on the knowledge users in the same way that a longitudinal design may have. Tracking the knowledge user’s experiences over the course of the project may have offered a more precise account of the impact of the various approaches to supporting HIT safety and the progression of the partnerships within the research [52]. Future research should focus on assessing the mechanisms by which the impact is achieved to articulate an optimal, replicable approach to knowledge mobilization. In addition, this study was situated in a nonacute setting, and therefore, the applicability of our findings is limited as such. However, given the adaptable and codeveloped nature of the processes for learning, it is possible that other health care settings may benefit from using similar approaches [45,85].

### Conclusions

Overall, the findings of this study indicate that the evidence-based, experiential learning model used in this instance was an impactful approach to supporting HIT safety in the context of clinical informatics. Furthermore, the intensive focus on HIT safety resulted in increased knowledge and some evidence of group-level identity transformation related to clinical informaticians’ management of HIT safety events. An embedded researcher model can be an effective mechanism to support clinical informaticians in learning and applying HIT safety practices in their work.

## Appendix

Multimedia Appendix 1 Semistructured interview guide.

## Abbreviations

EHR: electronic health record
HIT: health information technology
