# Supplementary material for: Clinical Informatics Team Members’ Perspectives on Health Information Technology Safety After Experiential Learning and Safety Process Development: Qualitative Descriptive Study
Source: JMIR Form Res. 2024 Feb 5;8:e53302. doi: 10.2196/53302 (PMC10877498; doi:10.2196/53302)
Supplement: Multimedia Appendix 1 [file formative_v8i1e53302_app1.pdf]

Clinical Informatics Team Members' Perspectives on HIT Safety After Experiential Learning and Safety Process Development: A Qualitative Descriptive Study

Focus Group Question Framework

1. Before we started this work – what were your thoughts about technology safety? How has this changed, if at all?
2. With regard to technology safety, what do you know now that you didn't know before – on a personal level? On an organizational level?
3. If at all, how has your awareness of technology safety impacted how you will do your work in the future?
4. Describe your experiences and thoughts about reviewing PSLS events, participating in safety huddles, and/or learning sessions?
    - What are some actual or potential safety concerns related to technology that you think are important to watch out for?
5. What do you think are the most important knowledge, skills, and attributes needed to promote safety in clinical informatics?
    - For example, what might be included in a course? Or onsite education?
6. Additional question if time permits: What's the most important thing about this work from your perspective?
